# National outbreak of *Pseudomonas aeruginosa* associated with an aftercare solution following piercings, July to September 2016, England

**DOI:** 10.2807/1560-7917.ES.2018.23.37.1700795

**Published:** 2018-09-13

**Authors:** Hannah Evans, Hikaru Bolt, Ellen Heinsbroek, Bryony Lloyd, Peter English, Samia Latif, Nicola Elviss, Jane Turton, Peter Hoffman, Paul Crook, Richard Puleston, on behalf of the Outbreak Control Team

**Affiliations:** 1 United Kingdom Field Epidemiology Training Programme, Public Health England, London, United Kingdom; 2 Field Epidemiology Services, National Infection Service, Public Health England, London and Nottingham, United Kingdom; 3 European Programme for Intervention Epidemiology Training (EPIET), European Centre for Disease Prevention and Control (ECDC), Stockholm, Sweden; 4 These authors contributed equally to this work; 5 Public Health England South East, Horsham, United Kingdom; 6 Public Health England East Midlands, Nottingham, United Kingdom; 7 Food Water and Environmental Microbiology Laboratory, National Infection Service, Public Health England, London, United Kingdom; 8 Antimicrobial Resistance and Healthcare Associated Infections Reference Unit, National Infection Service, Public Health England, London, United Kingdom; 9 University of Nottingham, School of Medicine, Division of Epidemiology and Public Health, Nottingham, United Kingdom; 10 The members of the Outbreak Control Team have been listed at the end of this article

**Keywords:** *Pseudomonas aeruginosa*, outbreaks, bacterial infections, body piercing

## Abstract

We report a national *Pseudomonas aeruginosa* outbreak from a common source following piercings between July and September 2016 in England. The multi-agency outbreak investigation included active case finding, microbiological testing of environmental samples and case specimens including Variable Number Tandem Repeat (VNTR) typing and a retrospective cohort study. Overall, 162 outbreak cases (29 confirmed, 14 probable and 119 possible) and 14 non-outbreak cases were identified; all confirmed cases had ear piercings (93% cartilage). Outbreak cases were predominantly female (95%) and had a median age of 18 years (interquartile range: 13–56 years). Nineteen outbreak cases required surgery under general anaesthetic The same outbreak VNTR type (11,3,5,3,3,3,6,4,7) was isolated from bottles of an aftercare solution from a single manufacturer and in specimens from confirmed cases who attended eight different piercing studios supplied with this product. In the cohort study, use of aftercare solution was associated with becoming a case (aOR: 4.60, 95% confidence interval: 1.65–12.90). Environmental, microbiological and epidemiological investigations confirmed that contamination during production of aftercare solution was the source of this national outbreak; highlighting challenges in the regulation of a cosmetic products used in the piercing industry and that guidance on piercing aftercare may need to be reviewed.

## Background

*Pseudomonas aeruginosa* is a Gram-negative bacterium commonly found in water and a variety of wet environments [1]. Between August 2012 and December 2013, *P. aeruginosa* accounted for 3.5–11.0% of bacterial skin and skin-structure infections among hospitalised patients in 35 hospitals in the United Kingdom (UK) [2]. Sporadic *Pseudomonas* infections following body piercings are recognised but common source outbreaks are rarely reported [3,4]. Previous *P. aeruginosa* infections and outbreaks have been linked to exposure of the piercing wound to fresh water, swimming pools and use of a contaminated cleaning solution during the piercing procedure [3,5-7]. A cross-sectional study in England estimated the prevalence of body piercing in adults at 10% [8]. Among those aged 16 to 24 years, 31% of body piercings resulted in complications, with 15% of piercings requiring professional help (i.e. from the piercer or healthcare professionals) and 0.9% of piercings resulting in hospital admission. Currently, no surveillance system for post-piercing infections exists in the UK.

### Identification of the outbreak

On 31 August and 01 September 2016, the Public Health England (PHE) East Midlands and Surrey and Sussex Health Protection Teams (HPTs), South East England, were alerted by two local hospitals to a cluster of six and eight cases of ear abscesses, respectively, following piercings at two piercing studios located 150km apart. Four cases were confirmed with *P. aeruginosa* at each hospital site. Review of laboratory surveillance data indicated no other exceedances of *P. aeruginosa* in East Midlands, South East England or nationally suggesting that the clusters could be associated with the two piercing studios rather than part of a wider outbreak.

A multi-agency national outbreak control meeting was convened on 8 September 2016 to investigate and prevent further infections. Due to the large distance between the two piercing studios, investigations focused on identifying if the causative strain was common to both piercing studios and if so, whether there was any common source exposure. An aftercare solution, referred to hereafter as aftercare solution X, produced by a single manufacturer in the East Midlands with nationwide distribution was rapidly identified as the probable source. Initial microbiological investigations identified a common Variable Number Tandem Repeat (VNTR) typing profile (11,3,5,3,3,3,6,4,7) of *P. aeruginosa* from isolates from confirmed cases attending either of the two piercing studios.

We describe the investigation and control measures taken for this national *P. aeruginosa* outbreak.

## Methods

### Case definitions

A sensitive case definition was used for case finding, which did not specify attendance at piercing studios supplied with aftercare solution. Following strong microbiological evidence linking aftercare solution X with infection, case definitions were subsequently revised to accurately describe the extent of this outbreak, by distinguishing between outbreak and non-outbreak cases as shown in Box 1.

Box 1 ***Pseudomonas aeruginosa* outbreak case definitions, July–September 2016, England**

**Confirmed outbreak case:** a person who had a laboratory confirmed *P. aeruginosa* infection with the outbreak Variable Number Tandem Repeat (VNTR) type (11,3,5,3,3,3,6,4,7) following a piercing (any part of the body), anywhere in England, between 1 July 2016 and 30 September 2016.

**Probable outbreak case:** a person who had a laboratory-confirmed *P. aeruginosa* infection for which no VNTR typing was available, following a piercing (any part of the body), conducted at a studio known to have been supplied with aftercare solution X, between 1 July 2016 and 30 September 2016.

**Possible outbreak case:** a person who had a piercing (any part of the body) conducted at a studio known to have been supplied with aftercare solution X between 1 July 2016 and 30 September 2016 with a self-reported infection at the site of the piercing that required antibiotics and/or surgical drainage and/or developed pus discharge, for which no laboratory confirmation was available.

**Non-outbreak case:** a person who had a laboratory confirmed *P. aeruginosa* infection with a type different from the outbreak VNTR type (11,3,5,3,3,3,6,4,7) following a piercing (any part of the body) conducted anywhere in England, OR where no VNTR typing was available, following a body piercing (any part of the body) conducted at a studio known not to have been supplied with aftercare solution X, between 1 July 2016 and 30 September 2016.

**Exclusion criteria:** a person who had a sample which tested negative for *P. aeruginosa* OR for whom no information about the piercing studio was available OR for whom no laboratory confirmation was available and who attended a studio not known to have been supplied with aftercare solution X.

### National case finding

A briefing note was issued to PHE staff including HPTs throughout England and sent to relevant authorities of the devolved administrations. A patient safety notification was also issued to all General Practitioners, Ear Nose & Throat Departments and Accident and Emergency departments in England. Clinicians were asked to consider the possibility of *P. aeruginosa* in patients with piercing-site infections, provide appropriate treatment, and to report confirmed cases to their local HPT. Cases reported to HPTs were interviewed using a standardised questionnaire to gather information on the piercing studio, type of piercing, the process used in the studio, aftercare advice and practice, products provided and if medical care for a post-piercing complication was sought.

### Retrospective cohort study in two piercing studios, South East England and East Midlands

To test the hypothesis that aftercare solution X was associated with infection, we conducted a retrospective cohort study in the two piercing studios where the outbreak was first identified. Individuals aged 16 years or older who had a body piercing between 1 July and 10 September 2016 at either South East England or East Midlands piercing studios were included. An online questionnaire was emailed to piercing studio clients on 3 October 2016 (South East England) and 17 November 2016 (East Midlands). The questionnaire included questions on patient details, demographics, clinical history, use of medical services post-piercing, piercing details, use of aftercare solution X and potential water/aqueous exposures e.g. swimming pools.

Different case definitions were used for the cohort study (Box 2). Cohort case definitions were piercing-based as individuals could report multiple piercings during the study period, in contrast to the person-based case definitions as described in Box 1. Confirmed cases were identified by cross-matching data with the case finding line list and national laboratory surveillance data. Individuals who provided limited information or were confirmed with an infection other than *P. aeruginosa* were excluded.

Box 2 ***Pseudomonas aeruginosa* outbreak cohort case definition, July–September 2016, England**

**Cohort case definition:** a piercing (any part of the body) conducted at the East Midlands or South East England piercing studios between 1 July 2016 and 10 September 2016, in a person aged 16 years or older with a self-reported infection at the site of the piercing that required antibiotics and/or surgical drainage and/or developed pus discharge.

We performed univariable and multivariable analysis using Pearson’s chi-squared test, Fisher’s exact test and logistic regression, as appropriate. Variables with a p value < 0.2 and a priori variables age, sex and cartilage piercing were considered for inclusion in the model using a backwards stepwise approach. We identified a final model using the likelihood ratio test and observing changes in odds ratios (ORs) to assess for plausible confounding and interactions between the most strongly associated variables.

Dose-response relationships of daily frequency and duration of aftercare solution X used were explored using a score test for linear trend, if appropriate [9]. A *post-hoc* sensitivity analysis was performed using a more specific case definition by excluding cases reporting a shorter duration of symptoms (i.e. pus discharge < 8 days).

### Environmental investigations

Environmental health officers (EHOs) visited the East Midlands and South East England piercing studios and with Trading Standards (a local government service that performs routine inspections based on consumer complaints, with the aim to support businesses and consumers) visited the manufacturing site of aftercare solution X. At the piercing studios, EHOs reviewed piercing procedures and sampled common products in use including environmental cleaning solutions, skin puncture needles, jewellery, piercing equipment and bottles (opened/unopened) of aftercare solution. At the manufacturing site of the aftercare solution X, EHOs reviewed the production process, sampled equipment and materials used in the manufacturing process and examined documentation to determine the extent of the distribution chain. Environmental samples were sent to PHE Food, Water and Environmental (FWE) Microbiology Laboratories. Samples were handled based on methods of The Microbiology of Drinking Water (2010) – Part 8- Method for the Isolation and Enumeration of *Aeromonas* and *P. aeruginosa* by membrane filtration [10]. All environmental samples with presumptive *P. aeruginosa* were sent to the PHE Antimicrobial Resistance and Healthcare Associated Infections (AMRHAI) unit for confirmation and typing using VNTR analysis [11,12].

### Microbiology investigations

Samples from affected cases were tested for *P. aeruginosa* by local NHS microbiology laboratories and isolates were sent to the AMRHAI reference unit for confirmation and typing by VNTR [11,12]. Antibiotic susceptibility testing was by agar dilution and results interpreted using European Committee on Antimicrobial Susceptibility Testing (EUCAST) breakpoints (version 8.0) [13].

## Results

### Descriptive analysis

**Figure 1:**
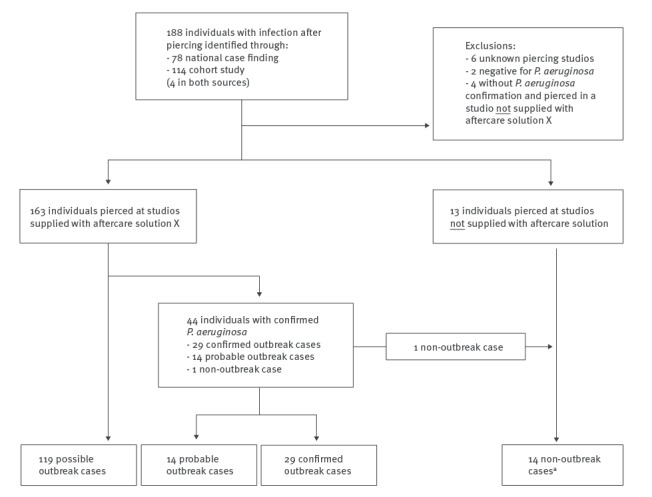
Individuals with infection following body piercing, England, July–September 2016 (n = 188)

^a^14 non-outbreak cases were identified who attended studios not known to be supplied with aftercare solution X (n = 13) and/or had a confirmed *Pseudomonas aeruginosa* infection with a different Variable Number Tandem Repeat (VNTR) strain from the outbreak cases (and were all distinct from each other) (n = 9).

In total, 188 individuals with infection following body piercing were identified during this outbreak (Figure 1); 162 outbreak cases (29 confirmed, 14 probable and 119 possible) and 14 non-outbreak cases. Twelve individuals were excluded.

**Figure 2:**
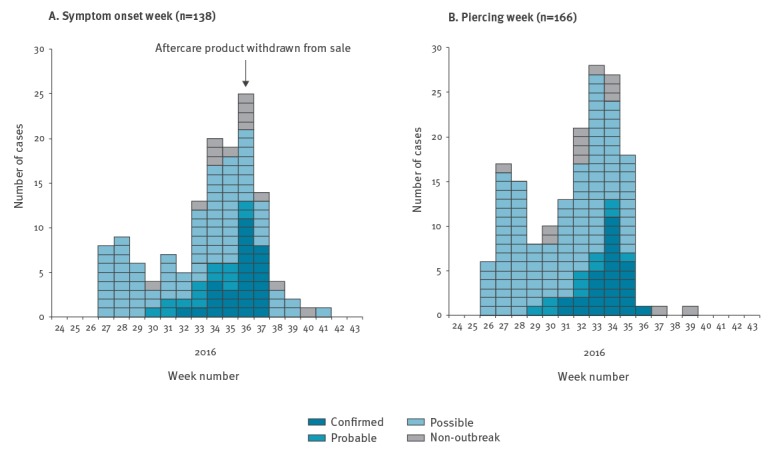
Distribution of cases of infection by week of (A) symptom onset and (B) piercing, weeks 24–43, England, 2016^a-d^

^a^ Cohort study ended on week 36; therefore, a decrease in number of cases as shown in (A) was expected.

^b^ Week 24 started on 13 June 2016.

^c^ Piercing week was missing for 10 cases.

^d^ Where onset week was unavailable, week attended primary care, hospital or sample taken (whichever closest to piercing date) was used where possible.

Outbreak cases were predominantly female (154/162, 95%) and had a median age of 18 years (interquartile range (IQR) 13–56 years) (Figure 2). All 29 confirmed cases were ear piercings (93% cartilage, 7% ear site unknown).

**Figure 3:**
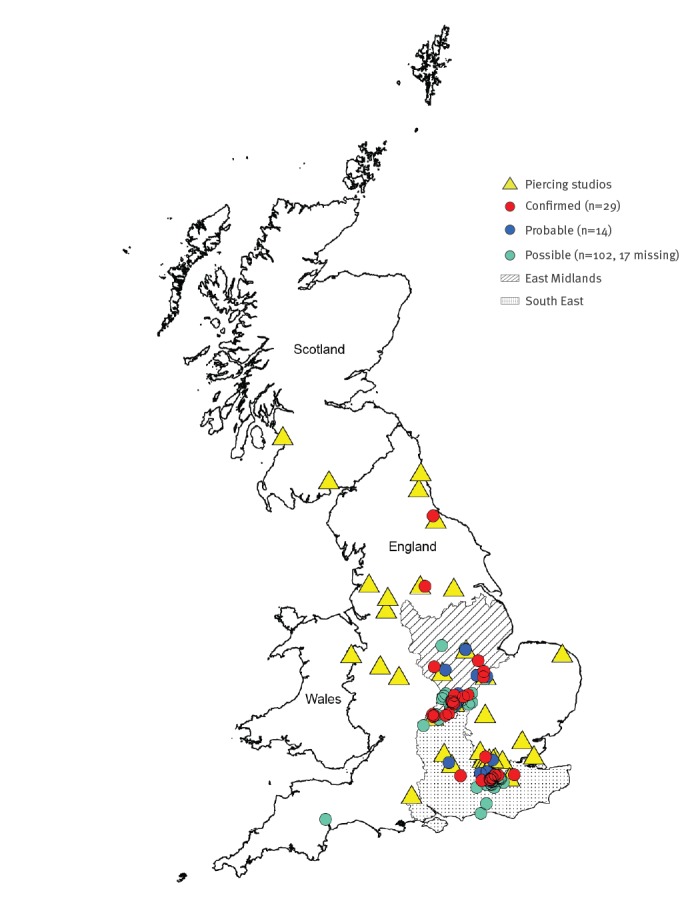
Geographical distribution of 38 piercing studios in receipt of aftercare solution X with confirmed^a^, probable^b^ and possible^c,d^ cases, England and Scotland, July–September 2016 (n = 145)

Aftercare solution X was supplied to at least 38 piercing studios (36 in England, two in Scotland). Of these, 10 gave rise to outbreak cases. Confirmed and probable cases were identified across England predominantly in South East England (n = 19) or East Midlands (n = 15) where the outbreak was first identified. No cases were identified in Scotland (Figure 3).

Contains Ordinance Survey data © Crown copyright and database right 2018. Contains Royal Mail data © Royal Mail copyright and database right 2018. Contains National Statistics data © Crown copyright and database right 2018.

19 confirmed and probable cases in the South East, and 15 confirmed and probable cases in the East Midlands.

Among outbreak cases where information was known, 58/147 (39%) attended primary care, 52/153 (34%) attended hospital and 44/152 (29%) required a medical procedure; 19/147 (13%) required surgery under general anaesthetic, 20/147 (14%) required a medical procedure without general anaesthetic and 2/149 (2%) required a medical procedure of unknown nature. Information on medical procedures was incomplete for 17 cases.

### Retrospective cohort study in two piercing studios, South East England and East Midlands

A total of 1,237 individuals were sent the online questionnaire, with 18% (n = 222) responding, reporting a total of 235 piercings between them. Of these 222 individuals, 114 individuals met the cohort case definition (Box 2) and were included in the cohort (attack rate: 49% among piercings). Of the 114 individuals, five cases were confirmed with *P. aeruginosa* infection of which two were confirmed with the outbreak VNTR type and three had no VNTR typing data available. Thirteen respondents reported two piercings during the study period.

Most respondents (212/222) and cases (110/114) were female. Median age of respondents was 21 years (IQR: 18–30 years). No difference in age (Wilcoxon rank-sum test, p value = 0.14) or sex (Fisher’s exact test, p value = 0.53) between cases and non-cases was identified. Among cases, 51 (45%) were ear cartilage piercings, 16 (14%) were ear lobe piercings and 47 (41%) were non-ear piercings. Cases most commonly reported pus discharge (n = 105; 92%) at the piercing site, with more than two weeks the most frequently reported duration (28%).

In the final multivariable model, use of aftercare solution X (aOR 4.60; 95% confidence interval (CI) 1.65–12.87, p value < 0.01), and any cream use (aOR 5.89; 95% CI: 1.21–28.62, p value = 0.01) were strongly associated with becoming a case (Table 1); younger age was also found to be associated with becoming a case. No dose-response relationships were detected between the use of aftercare solution X and becoming a case. The presence of an interaction between any cream use and aftercare solution X could not be explored due to small numbers.

**Table 1:** Univariable and multivariable associations between exposures and infection following body piercing among clients at two piercing studios, ranked by ascending aOR, East Midlands and South East England, July–September 2016 (n = 235 piercings, unless otherwise indicated)

Exposure	Total	Cases	AR (%)	Univariable analysis	Multivariable analysis	p value
				OR (95% CI)	aOR^d^ (95% CI)	
**Cartilage piercing**						
No	119	63	53%	Reference	Reference	NA
Yes^a^	116	51	44%	0.70 (0.42–1.17)	0.72 (0.40–1.29)	0.27
**Age**	**NA**	**NA**	**NA**	**0.97 (0.95-1.00)**	**0.97 (0.95-1.00)**	**0.02**
**Gender**						
Male	10	4	NA	Reference	Reference	NA
Female	225	110	49%	1.43 (0.39-5.22)	1.89 (0.43-8.20)	0.39
**Piercing tool (n = 223)**						
Piercing needle	201	92	46%	Reference	Reference	NA
Piercing gun	22	16	NA	3.16 (1.19–8.40)	2.22 (0.78–6.33)	0.12
**Aftercare solution X use**						
No	28	5	NA	Reference	Reference	NA
Yes	207	109	53%	5.12 (1.87–13.98)	4.60 (1.65–12.87)	0.001
**Cream use^c^**						
No	221	102	46%	Reference	Reference	NA
Yes	14	12	NA	7.00 (1.53–32.01)	5.89 (1.21–28.62)	0.01
**Other saline solution use**						
No	220	108	49%	Reference	NA	NA
Yes	15	6	NA	0.69 (0.24–2.01)	NA	NA
**Homemade saline use**						
No	163	77	47%	Reference	NA	NA
Yes	72	37	51%	1.18 (0.68–2.06)	NA	NA
**Soap use**						
No	220	107	49%	Reference	NA	NA
Yes	15	7	NA	0.92 (0.32–2.64)	NA	NA
**Antibacterial solution use**						
No	202	98	49%	Reference	NA	NA
Yes	33	16	NA	1.00 (0.48–2.09)	NA	NA
**Piercing site sprayed before/during procedure (n = 156)**						
No	92	45	49%	Reference	NA	NA
Yes	64	33	52%	1.11 (0.59–2.10)	NA	NA
**Time started using aftercare solution X (n = 233)**						
No aftercare solution X use	28	5	NA	Reference	NA	NA
Same day as piercing	177	92	52%	4.98 (1.81–13.68)	NA	NA
One or more days after piercing	28	15	NA	5.31 (1.57–17.97)	NA	NA
**Daily frequency of aftercare solution**						
No aftercare solution X use	28	5	NA	Reference	NA	NA
Once a day	20	11	NA	5.62 (1.52–20.80)	NA	NA
Twice a day	118	54	46%	3.88 (1.38–10.90)	NA	NA
3 times a day	53	35	NA	8.94 (2.91–27.46)	NA	NA
4 times or more a day	16	9	NA	5.91 (1.48–23.56)	NA	NA
**Duration of aftercare solution X use (weeks; n = 230)**						
No aftercare solution X use	9	1	NA	Reference	NA	NA
Up to 1 week	28	16	NA	10.67 (1.17–97.18)	NA	NA
Up to 2 weeks	50	26	NA	8.67 (1.01–74.52)	NA	NA
Up to 3 weeks	35	21	NA	12.00 (1.35–106.80)	NA	NA
Up to 4 weeks	27	11	NA	5.50 (0.60–50.44)	NA	NA
More than 4 weeks	81	35	43%	6.09 (0.73–50.96)	NA	NA
**Type of piercing (n = 232)**						
Bar	101	54	53%	Reference	NA	NA
Stud	90	43	48%	0.80 (0.45–1.41)	NA	NA
Other	41	16	NA	0.56 (0.27–1.17)	NA	NA
**Exposure to water**						
No	191	92	48%	Reference	NA	NA
Yes	44	22	NA	1.08 (0.56-2.07)	NA	NA
**Exposure to hot tub**						
No	231	112	48%	Reference	NA	NA
Yes	4	2	NA	1.06 (0.15–7.67)	NA	NA
**Exposure to open water**						
No	215	105	49%	Reference	NA	NA
Yes	20	9	NA	0.86 (0.34-2.15)	NA	NA
**Exposure to swimming pool**						
No	197	94	48%	Reference	NA	NA
Yes	38	20	NA	1.22 (0.61–2.44)	NA	NA
**Application of aftercare solution X (n = 205)**						
Indirectly onto piercing site only	53	29	NA	Reference	NA	NA
Directly onto piercing site only	116	61	53%	0.92 (0.48–1.76)	NA	NA
Indirectly and directly onto piercing site	36	18	NA	0.83 (0.35-1.93)	NA	NA

aOR: adjusted odds ratio; AR: attack rate; CI: confidence interval; NA: not applicable; OR: odds ratio.

^a^ Cartilage piercing includes antihelix, anti-tragus, conch, daith, helix, rook or tragus piercings, or where individual indicated a cartilage piercing. Non-cartilage piercing includes earlobe piercings or non-ear piercings, e.g. nose, navel, tongue.

^b^ At least five different products reported.

^c^ Exposure to water corresponds to any exposure to hot tub, open water or swimming pool.

^d^ Based on 223 observations and adjusted for age, sex, aftercare solution X use, cream use, piercing gun and cartilage piercing.

Among the 114 cohort cases, 109 (96%) reported aftercare solution X use compared with 12 (11%) reporting any cream use, with no cases reporting cream use only. Nine cohort cases reported the use of different antibacterial/antiseptic creams and three reported the use of three different other creams. Median duration of aftercare solution X use did not significantly differ between cases and non-cases (20 days vs 15 days, p value = 0.23). In the sensitivity analysis (excluding cases reporting a shorter duration of symptoms (i.e. pus discharge < 8 days), similar results were obtained i.e. use of aftercare solution X (aOR: 6.03; 95% CI: 1.35–27.04) and any cream use (aOR: 6.50; 95% CI: 1.26–33.66).

### Environmental investigations

Infection prevention and control procedures were satisfactory at the East Midlands and South East England piercing studios. At the production site for the aftercare solution, environmental inspections identified inadequate quality control and documentation procedures. Aftercare solution X was a 100ml bottle of saline (0.9% sodium chloride) with the possible addition (upon request) of retail tea tree oil diluted with de-ionised water.

In total, 88 environmental samples were submitted for testing including bottles of aftercare solution X, jewellery, puncture needles, liquids used during the piercing procedure, and samples from the manufacturing site. Twenty-one environmental samples were positive for *P. aeruginosa* including unopened aftercare solution X bottles from studios (n = 10), opened aftercare solution X bottles from cases (n = 4) and environmental samples from the production equipment and materials at the manufacturing site of aftercare solution X (n = 7). High levels of *P. aeruginosa* (> 100 colony forming units (cfu) per ml) were isolated from all samples positive for *P. aeruginosa*. To put this in context, levels > 10 cfu per 100ml are considered unsatisfactory in augmented care ward hot and cold water systems in the hospital setting and in swimming pool waters [14,15].

### Microbiological investigations

The same outbreak VNTR type (11,3,5,3,3,3,6,4,7) was identified in the 21 environmental samples testing positive for *P. aeruginosa* and in the 29 confirmed outbreak cases who visited a piercing studio supplied with aftercare solution X. This VNTR type had only previously been identified in three of 18,755 isolates tested by AMRHAI since 2010 and is therefore very unusual. Representatives tested were susceptible to amikacin, gentamicin, piperacillin-tazobactam, ceftolozane/tazobactam, ceftazidime, imipenem, meropenem and colistin, with intermediate susceptibility to aztreonam, with intermediate susceptibility to aztreonam. The VNTR types for non-outbreak cases were all distinct from each other and from the outbreak strain.

### Control measures

On 9 September 2016, the aftercare solution X manufacturer was issued with a notice to cease manufacture and supply of the product and subsequently voluntarily recalled the product from sale. EHOs removed aftercare solution X from all piercing studios known to be supplied. Piercing studios supplied with aftercare solution X were requested to contact clients to advise them to cease using the product. Among cohort respondents, 70% (150/215) reported awareness of the recall of aftercare solution X, predominantly via environmental health (32%, 69/215) or the piercing studios (28%, 60/215). On 15 September 2016, a proactive public press statement was issued to advise the public to cease using the product and seek medical attention if required, advice that was reiterated during media interviews.

## Discussion

The investigation found strong environmental, microbiological, and descriptive epidemiological evidence and supporting analytical epidemiological evidence that the large national outbreak of *P. aeruginosa* infections following piercings in England during 2016 was caused by the use of aftercare solution X.

VNTR typing was central to this outbreak investigation by identifying a common outbreak VNTR profile shared between cases, environmental samples (taken from the manufacturing site), and directly from bottles of aftercare solution X (opened and unopened). The outbreak VNTR type was distinct from cases found to be unrelated to the outbreak i.e. pierced at studios not known to have been supplied with aftercare solution X.

The cohort study identified a four times higher odds of becoming unwell in individuals who used aftercare solution X compared with those who did not use aftercare solution X. Use of any cream was also identified as a risk factor for infection although it is unlikely that cream use was responsible for this outbreak. This is because: (i) only 11% of cases reported any cream use compared with 96% for aftercare solution X, (ii) the most commonly reported types of cream were antibacterial/antiseptic creams indicating they were likely used in response to an infection although information on time of cream application was not collected, (iii) cases reported using different brands/types of cream rather than a single type.

There have been previous outbreaks of *P. aeruginosa* infections linked to contaminated aftercare solutions [3,5-7]. During an outbreak in Oregon, United States (US), *P. aeruginosa* was cultured from a ‘single-use’ disinfectant spray bottle being refilled repeatedly with contaminated water from a sink and used to clean the piercing gun and earrings [3]. In New York, US, *P. aeruginosa* was also cultured from an aftercare solution made on site and given to customers at a piercing studio [5]. In both these outbreaks cartilage piercings were associated with developing *P. aeruginosa* infections. It is thought that upper ear cartilage piercings are susceptible to infection due to the lack of blood supply to the area and can be slow to heal [3,16]. In this outbreak, 27/29 (93%) of confirmed cases had an ear cartilage piercing, however, we found no association between cartilage piercings and infections in the analytical study. This may be due to only five laboratory confirmed *P. aeruginosa* cases participating in the survey and therefore may have reduced our ability to detect cartilage piercings as a risk factor. We found an unprecedented number of complications; 44 cases requiring medical procedures compared with at least five reported in the Oregon outbreak and 15 reported in the New York outbreak.

There were several limitations in this outbreak investigation. Initial case definitions used for case finding were broad to ensure the outbreak investigation identified all likely cases. However, as a result we identified a number of non-outbreak cases and in the absence of routine surveillance data, we were unable to indicate whether this was an expected background rate. This potentially inflated the initial number of possible outbreak cases and so a refined case definition was developed to describe the outbreak. Even with the refined case definition, it is likely sporadic infections following piercings were incorrectly reported as possible cases in this outbreak.

The questionnaire was disseminated more than 3 weeks following the recall of aftercare solution X from the market and several months following the original piercing dates which may have influenced their recall of information. In addition, the information sent to clients instructing them to stop using aftercare solution X was provided before the questionnaire was circulated; this likely influenced their responses. Despite these limitations, the findings from the cohort study were in line with the descriptive epidemiology, environmental and microbiological evidence, of the source being aftercare solution X and helped describe the high burden of infections amongst consumers.

Aftercare products such as aftercare solution X, implicated in this outbreak, are regulated by Trading Standards as cosmetics rather than medicines. Aftercare solution X was described on the label as ‘ideal for irrigation and wound cleansing after piercing’ and included a mark indicating a product that conformed to the legal standards of the European Economic Area (EEA). EU regulation (EC1223/2009) on cosmetic products states that it “should not endanger health and safety of consumers” and “should be produced according to good manufacturing practice” [17]. The regulation includes a requirement for manufacturers to produce a Cosmetic Product Safety Report, which must include information about the product’s microbiological quality. EU guidance (SCCS/1416/11) for the testing of cosmetics ingredients and their safety evaluation states that “*P. aeruginosa*…must not be detectable” [18]. There was no quality control of the manufacturing process or assessment of the product’s microbiological quality.

### Lessons learnt and future considerations

This outbreak investigation identified several non-outbreak cases of *P. aeruginosa* infections after piercing, which may indicate a wider issue and is consistent with a previous study where 31% of pierced clients experienced complications such as infection, bleeding, allergy, tear or injury [8]. Further research would be useful to gain a better understanding of the rate of infections following piercing and the associated risk factors

Since aftercare solution X had a mark indicating its compliance to EEA standards, it would have been plausible for the product to be distributed widely in Europe. There may be a case for greater regulation of the quality and safety of products used for wound cleansing in the piercing and tattoo industry in order to prevent potential European-wide outbreaks. As compliance to good manufacturing practice often seems less rigorously enforced for cosmetic products vs medicinal ones, this is an area where there needs to be a high degree of vigilance if outbreaks are suspected. Furthermore, this outbreak and the challenges in regulation, highlight that guidance on piercing aftercare may need to be reviewed with renewed scrutiny of the evidence on the benefits of using saline solution in post-piercing aftercare.

## Notes

**Members of the Outbreak Control Team:** Neil Anstey, Gail Beckett, Hikaru Bolt, Colin S Brown, André Charlett, Sarah Collins, Paul Cosford, Max Courteney-Pillinger, Derrick Crook, Paul Crook, Matthew Daft, Nicola Elviss, Peter English, Hannah Evans, Ellen Heinsbroek, Patricia Hennessy, Peter Hoffman, Sue Ibbotson, Rob Johnston, Dervla Kenna, Theresa Lamagni, Samia Latif, Bryony Lloyd, Jonathan Lloyd, Liz Loosemore, Helen Maguire, Ailsa Mcilwaine, Sophie Newitt, John Paul, Richard Puleston, Maria Saavedra-Campos, Russell Sinclair, David Spence, Jane Turton, Neville Verlander, Shaun Whelan, Julian Wilks, Caroline Willis, Amanda Wright.
